# Dengue risk assessment using multicriteria decision analysis: A case study of Bhutan

**DOI:** 10.1371/journal.pntd.0009021

**Published:** 2021-02-10

**Authors:** Tsheten Tsheten, Archie C. A. Clements, Darren J. Gray, Kinley Wangdi

**Affiliations:** 1 Australian National University, Canberra, Australia; 2 Royal Centre for Disease Control, Ministry of Health, Thimphu, Bhutan; 3 Telethon Kids Institute, Nedlands, Australia; 4 Curtin University, Perth, Australia; Australian Red Cross Lifelood, AUSTRALIA

## Abstract

**Background:**

Dengue is the most rapidly spreading vector-borne disease globally, with a 30-fold increase in global incidence over the last 50 years. In Bhutan, dengue incidence has been on the rise since 2004, with numerous outbreaks reported across the country. The aim of this study was to identify and map areas that are vulnerable to dengue in Bhutan.

**Methodology/Principal findings:**

We conducted a multicriteria decision analysis (MCDA) using a weighted linear combination (WLC) to obtain a vulnerability map of dengue. Risk factors (criteria) were identified and assigned with membership values for vulnerability according to the available literature. Sensitivity analysis and validation of the model was conducted to improve the robustness and predictive ability of the map. Our study revealed marked differences in geographical vulnerability to dengue by location and season. Low-lying areas and those located along the southern border were consistently found to be at higher risk of dengue. The vulnerability extended to higher elevation areas including some areas in the Capital city Thimphu during the summer season. The higher risk was mostly associated with relatively high population density, agricultural and built-up landscapes and relatively good road connectivity.

**Conclusions:**

Using MCDA, our study identified vulnerable areas in Bhutan during specific seasons when and where the transmission of dengue is most likely to occur. This study provides evidence for the National Vector-borne Disease Control programme to optimize the use of limited public health resources for surveillance and vector control, to mitigate the public health threat of dengue.

## Author summary

Dengue is an important vector-borne viral disease affecting humans. In Bhutan, dengue incidence is on the rise with increased frequency of outbreaks and spread to new areas. Outbreaks were reported from places as high as above 900m above sea level in recent years. However, dengue control activities in Bhutan are usually initiated at the time of outbreaks. This often leads to a large number of cases and overburden the health system. To address these issues, we developed dengue risk maps at a fine spatial resolution by combining risk factors that mediate the transmission of dengue using a weighted linear combination. Vulnerability to dengue was spatially heterogeneous and varied by season. Dengue is highly vulnerable in low-lying areas throughout the season. However, the vulnerability extended to higher geographical elevations including the nation’s capital during the summer season. The study provides a firm evidence-base to prioritize areas and seasons for dengue control strategies in Bhutan.

## Introduction

The global incidence of dengue has increased over the last few decades from over 8.3 million reported infections in 1990 [1] to approximately 80 million dengue cases in 2015 (representing a ten-fold increase) [2]. The number of deaths also increased from 12,300 in 2005 to 18,400 in 2015 [3]. Before 1970, only nine countries experienced severe dengue epidemics. Today, more than 120 countries have endemic dengue virus (DENV) transmission, with severe dengue resulting in 21,000 deaths annually [4]. There are now estimated 390 million dengue infections worldwide every year (95% credible interval [CrI] 284–528 million), of which 96 million (95% CrI 67–136 million) manifest clinically with varying degree of severity [5]. Dengue is the leading cause of hospitalization and death among children in the South East Asia region [6].

Implementing effective interventions for dengue involves identification of priority areas and times of the year that are most vulnerable to the disease. However, this presents a challenge in developing countries without adequate data [7]. Misdiagnosis and asymptomatic infections impede the detection of cases, resulting in delayed responses [5]. Moreover, surveillance systems do not provide adequate information for instituting informed dengue control activities, in particular, due to massive under-reporting [8, 9]. Identifying areas at risk of dengue is of paramount importance to prioritize resources and provide effective responses to dengue outbreaks [10].

In the 2019 dengue epidemic, dengue cases were reported from 19 of the 20 districts in Bhutan, including many districts that had never previously had dengue. Unfortunately, the current national surveillance system does not capture information on the source of infection, i.e. whether it was acquired locally or imported from other districts or countries, and so it is not known whether all cases of dengue were acquired in the district where they were reported. This information is crucial to understand the epidemiology of dengue for planning control activities. Therefore, methods need to be applied to understand whether particular locations are likely to sustain local dengue transmission.

Recent advances in geographical information systems (GIS) and remote sensing technologies have been increasingly used to study the spatial relationships between vector-borne diseases and the factors that influence their distribution [11]. These technologies have been implemented in several ways, including as the basis for spatial decision support tools to improve resource allocation for disease surveillance and control activities [12] and predicting high risk locations for these diseases [13]. A GIS-based multi-criteria decision analysis (MCDA) is one such decision support tool, offering a straightforward approach to conceptualizing the complex web of factors and interactions that mediate the transmission of disease [14]. MCDA integrates multiple factors and weighs their importance by incorporating knowledge and data from diverse sources [15, 16]. This can be operationalised in different ways; here, we used a Weighted Linear Combination (WLC) algorithm for combining information to produce a dengue vulnerability map.

The aim of this study was to generate dengue vulnerability maps for Bhutan for different seasons. These maps can be used to visualise dengue vulnerability of different areas for prioritizing limited public health resources for controlling dengue in Bhutan.

## Methods

### Ethics statement

Ethical clearance for this study was approved by the Research Ethics Board of Health (REBH), Ministry of Health, Bhutan (REBH/Approval/2020/033) and the Australian National University (ANU), Australia (2020/297).

### Study setting

**Fig 1:**
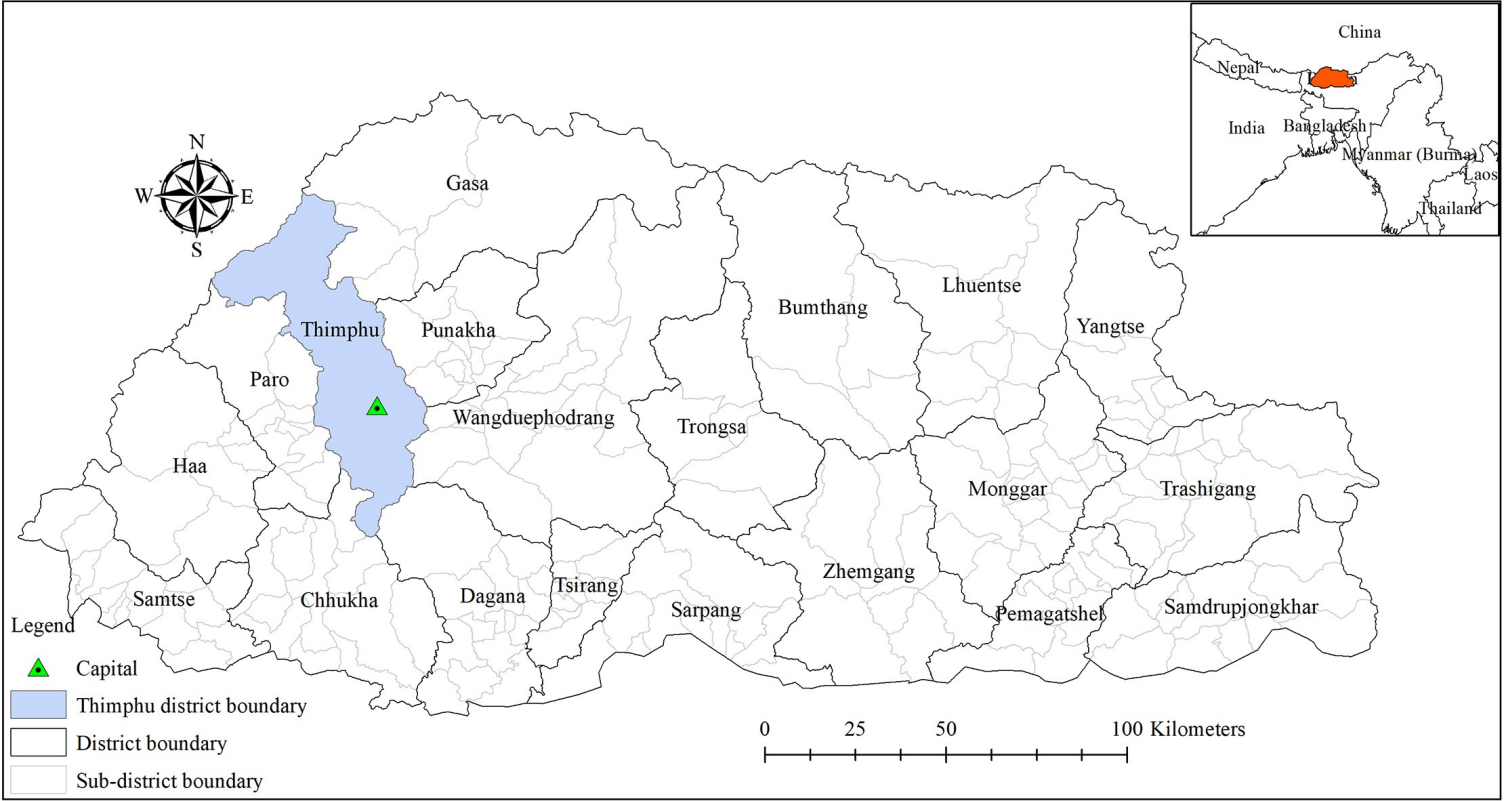
District (with name) and sub-district boundaries (in light grey colour) of Bhutan. The capital city (green triangle with dot inside) lies in Thimphu district, shown here in light blue.

Bhutan is one of Asia’s smallest nations, situated in the southern slopes of the eastern Himalayas between the People’s Republic of China in the North and India in the South, West and East. The country has a total area of 38,394 square kilometres and lies between 27° 30’ N and 90° 30’ E [17]. The country is administratively divided into 20 districts or *dzongkhags*, which are further divided into 205 sub-districts or *gewogs* (Fig 1).

Bhutan has a monsoonal climate with four seasons. The spring season starts in March and ends in May. The summer season, which commences in June and ends in August, is characterized by heavy rain and high temperatures. The rain continues into the autumn season, which stretches from September to November. From December, the winter sets in with frost covering much of the country, and it lasts until February [18].

### Study design

The MCDA process involves the following stages: 1) Defining the objective, 2) Identifying factors and constraints using different information sources (e.g. expert opinion, a literature search and analysis of historical data), 3) Defining the relationship between the factors and the vulnerability of a location for dengue, 4) Transforming or standardizing the values of the factors on a relative scale to allow comparison between each criterion, 5) Weighting the criteria on the basis of their relative importance to vulnerability, 6) Combining and aggregating all the layers/criteria to produce a final weighted estimate of vulnerability in each location and, 7) Conducting sensitivity analysis and validating the results.

### Objective

The objective of MCDA was to identify and prioritize areas for dengue control. Dengue control activities such as vector control in Bhutan are initiated only during outbreaks and when the health system is already disrupted. During an outbreak, healthcare facilities are overcrowded with limited space to care for patients, and staffing is insufficient to meet the patient load. To avoid such delays and provide prompt intervention measures, identification of areas at risk of dengue is important. Areas that are vulnerable for dengue might then be sites for sentinel or other targeted surveillance approaches, and protocols and systems can be developed in advance for mobilization of resources when and where they are required.

### Data sources

To validate the model, we used dengue cases between 2016–2019 from the National Early Warning, Alert, Response and Surveillance (NEWARS) database housed within the Ministry of Health of Bhutan. NEWARS is an integrated online disease surveillance system of all nationally notifiable diseases including dengue [19]. Dengue cases are defined as any patients presenting fever with any two of the following symptoms: headache, retro-orbital pain, rash, muscle/joint pain, positive tourniquet test and any warning signs of severe dengue, which include abdominal pain, persistent vomiting, mucosal bleeding, fluid accumulation, liver enlargement and elevated haematocrit value. These cases were further confirmed by rapid diagnostic test for dengue virus non-structural protein 1 (NS1), anti-dengue IgM and IgG antibodies. Only confirmed cases were reported into the NEWARS. Average annual dengue incidence in the districts varies between less than three cases to more than 21 cases per 10,000 inhabitants (S1 Fig). Dengue is seasonal with maximum dengue incidence reported during summer than other seasons (S1 Table).

Past studies have demonstrated multiple factors that influence the occurrence of dengue (S2 Table). We included temperature, rainfall, land use, population density, distance from the road network and the open water bodies/major rivers, upon which we base the vulnerability assessment.

Maximum temperature and rainfall variables were obtained from Worldclim (www.worldclim.org). These raster layers were developed at a spatial resolution of 30 seconds (~1 km^2^) between 1970 and 2000 [20]. In this study, seasonal averages were generated for both rainfall and temperature for the following time periods: a) December, January and February for the winter season; b) March, April and May for the spring season; c) June, July and August for the summer season; and, d) September, October and November for the autumn season. As elevation has been already used as a covariate in Worldclim data production, it was not used in the model construction [21].

Population density was obtained from the Worldpop (www.worldpop.org) global high-resolution population denominators project, which provides gridded population counts at 100 m spatial resolution (3-arc seconds) [22].

Land use data were obtained as a vector layer from the Ministry of Agriculture and Forestry, Royal Government of Bhutan. This was the latest data available for land use in the country. This layer has 20 categories, including many specific land cover types. The layer was re-categorized into three categories based on relevance to the vulnerability of dengue transmission: built-up (areas occupied by humans), agricultural land and others (natural environment. This vector layer was converted into a raster layer using the *conversion tool* in ArcGIS.

Similarly, the road network data was obtained from the Ministry of Work and Human Settlement as a vector layer. Euclidean distance to the nearest road was computed throughout Bhutan using the *distance* tool in ArcGIS. Finally, we considered the distance to the open water bodies/major rivers, obtained from the National Land Commission, Bhutan. Euclidean distance to the nearest open water was computed following similar steps as the road network.

**Fig 2:**
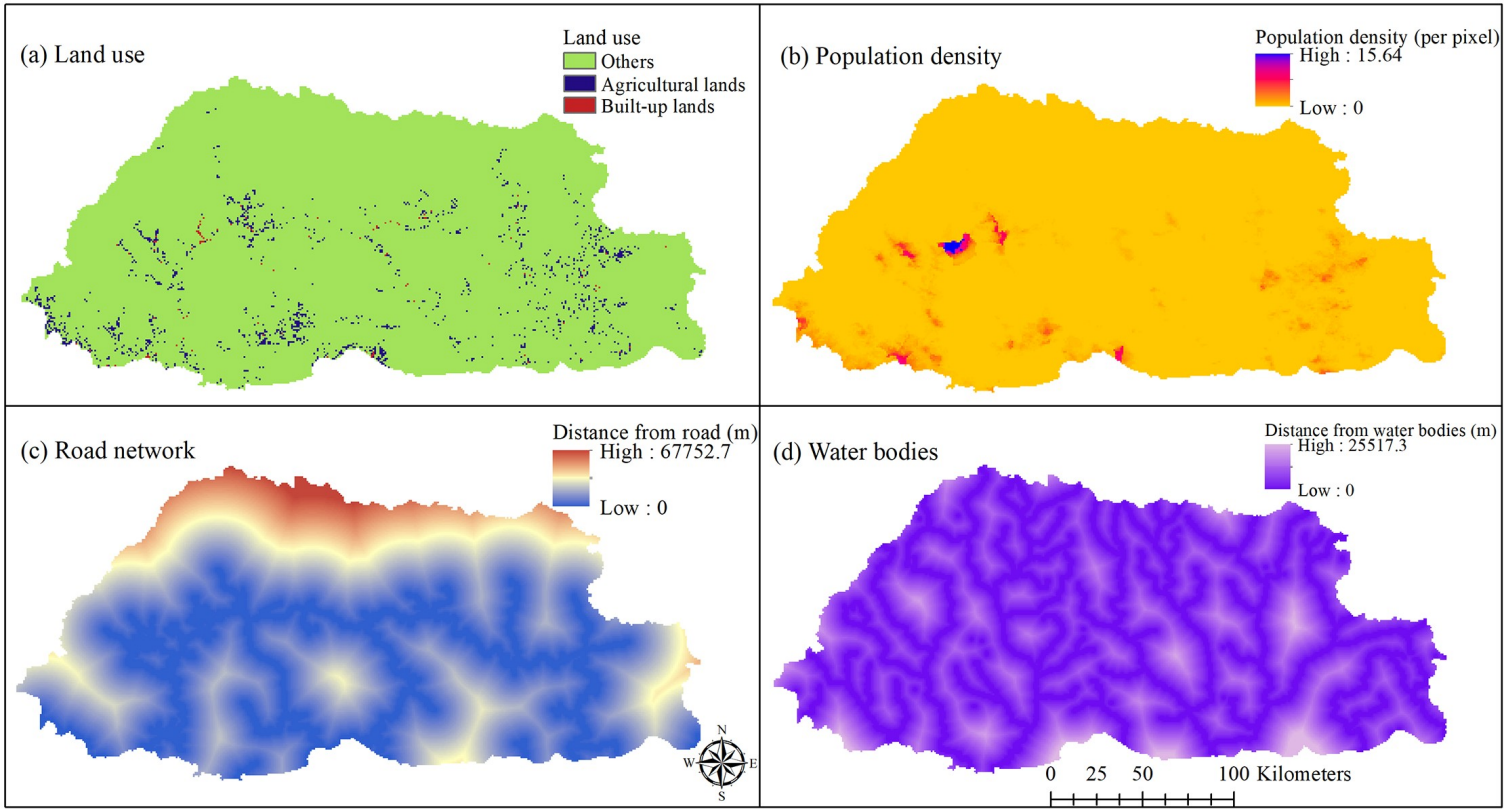
Patterns of land use types, distance from the road network (~1 decimal degrees = 111,000meters) and population density (number of people per pixel) in Bhutan.

**Fig 3:**
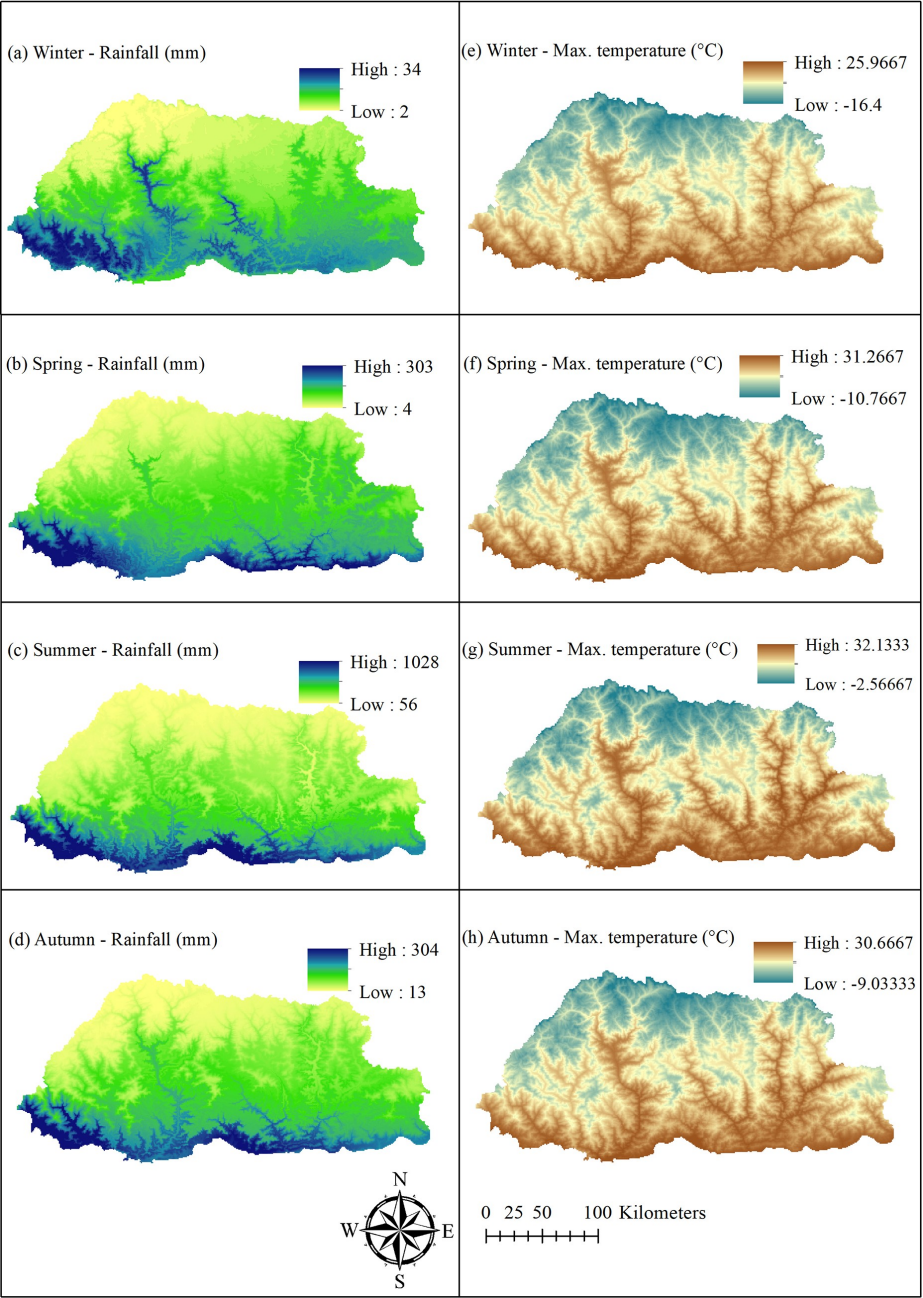
Patterns of seasonal average rainfall (left panel maps) and average maximum temperature (right panel maps) in Bhutan. Seasonal average data was computed by averaging three months for each season: Winter (December-February), Spring (March-May), Summer (June-August) and Autumn (September-November).

As data obtained from different sources were at diverse spatial scales, they had to be converted into uniform scales. All rasters were uniformly standardized at a pixel dimension of 0.0083 decimal degrees (which equals approximately 1km at the equator) covering the entire geographical extent of Bhutan. The land use and water bodies layers were reprojected from Geographic Coordinate System (GCS) Drukref_03 Transverse Mercator to the World Geodetic System (WGS) 1984 for spatial analysis. Raster maps were produced for all risk factors using this standardized spatial resolution and the same pixel size (Figs 2 and 3).

### Standardization of criteria (Risk factors) using membership functions

There exist different ways of standardizing or normalizing criteria such as Boolean constraints, linear transformation, value/utility functions and membership functions (MF). Rescaling factors into a standardized continuous scale allowed them to be compared and combined. In this study, fuzzy logic MF was used to determine the relationship between each factor and dengue vulnerability. Fuzzy set membership is the probability of an element belonging to a class (here, vulnerable/not vulnerable) in which boundaries between classes are not distinct. Using this approach can improve the accuracy of the output as the membership score represents a zone of gradual transition rather than a hard transition. The graded values ranged from 0 (i.e. complete non-membership or non-vulnerable in a fuzzy set) to 1 (i.e. complete membership or highly vulnerable in a fuzzy set).

### Temperature

Climatic conditions, including temperature and rainfall, play a major role in the local spread of dengue infection by directly affecting the life cycle, feeding activity, biting rates and incubation period of the disease [23]. Vector populations increase through processes such as the laying of eggs, egg hatching and growth of larva and pupa, all of which are favoured by higher temperatures. Higher temperatures reduce the extrinsic incubation period of DENV in *Aedes* mosquitoes and promote viral transmission [24, 25]. Furthermore, higher temperatures increase the speed of blood meal digestion and thus boost the feeding habits of mosquitoes [26]. The longevity of *Aedes* mosquitoes increases with an increase in temperature, with the optimum survival temperature ranging from 27–30°C, while temperature below 17°C and above 34°C are associated with low mosquito activity and thus lower transmission [25, 27]. Thus we input a symmetric relationship between vulnerability and temperature, with vulnerability rising from 17°C (below which the environment is not vulnerable), peaking at 27–30°C, and falling to 34°C (above which the environment is also not vulnerable) (Table 1).

**Table 1:** Parameters defining membership functions for dengue vulnerability in Bhutan.

Risk factors	Function shape	Not vulnerable	Maximum vulnerability	Rationale
Temperature (°C)	Symmetric	<17 & >34	27–30	Optimum surviving temperature for vector ranges from 27–30°C, while temperature <17°C & >34°C are associated with low mosquito activity & lower transmission
Monthly rainfall (mm)	Monotonic increasing (Linear)	<10 mm	>167 mm	Total monthly rainfall of 167mm is suitable for stable dengue transmission
Population density (per pixel)	Monotonic increasing (Linear)	< 1	>16	Populations >1 person per pixel are required for dengue transmission & vulnerability increases linearly to a maximum at 16 people per pixel
Distance from the road network (m)	Monotonic decreasing (Linear)	>5000m	<500m	Most dengue cases occur within 500m of a road, whilst dengue rarely occurs >5000m from the road network
Land use types	Categorical data	Natural environment	Built-up areas	Built-up areas are associated with highest dengue incidence followed by agricultural land. Natural environments such as forest, snow and barren land are not associated with dengue
Distance from the water bodies	Monotonic decreasing (Linear)	>20,000m	<500m	Most dengue cases occur within 500m of water bodies, whilst dengue rarely occurs >20,000m from water bodies

### Rainfall

Increased rainfall facilitates vector population growth by providing water required for mosquito breeding [28]. The impact of rainfall on vector density and consequent dengue infection varies across different geographical regions. Cheong et. al had reported the highest dengue incidence at a bi-weekly total rainfall between 215–302 mm in Malaysia [29]. In Taiwan, the relative risk of dengue was found to be significantly correlated with precipitation up to a maximum of 350mm/day at 70 days lag [30], while the peak dengue risk had been reported at a monthly cumulative rainfall of 720mm in China [31]. Countries in the World Health Organization South-East Asia Region (WHO-SEAR) including Bhutan are considered to have stable *Ae*. *aegypti* populations in geographical locations that receive an annual rainfall of greater than 2000mm [7]. Based on this relationship, dengue incidence varies seasonally (following a Sigmoidal function) with maximum incidence evident the month following a monthly cumulative rainfall of 167mm. Vulnerability in the current study was, therefore, assumed to increase monotonically with monthly rainfall above 10mm [32] (Table 1).

### Population density

High population density and uncontrolled urbanization have created ideal conditions for increased transmission of dengue [33]. Dengue incidence is assumed to increase linearly with increasing population density. Areas with population density <1 person per square km were standardized to zero, while areas with the maximum population density (i.e., 16) were standardized to one. A simple linear increasing function was used to describe this relationship between vulnerability and population density (Table 1).

### Land use

*Ae*. *aegypti* are commonly found around human dwellings, where female mosquitoes frequently and almost exclusively bite human hosts [26]. Therefore, this mosquito is predominantly found in high-density urban areas, and is rarely found in vegetated and forested land [34]. An abundance of *Ae*. *aegypti* is also associated with a high coverage of agricultural land use such as crops and paddy fields in some areas [35, 36]. Fragmentation of natural habitats can increase the local temperature and increase human-mosquito interactions, favouring the transmission of dengue [37]. On the continuous scale from 0 to 1, built-up areas were considered highly vulnerable and rated as “1”, agricultural lands were rated as “0.50”, while all others areas were considered not vulnerable to dengue and rated as “0”.

### Distance from the road network

Using the road network as a measure of urbanization, studies have reported a positive relationship between the distribution of dengue epidemics and density of the road network. Well-developed road networks facilitate the movement of people and increase the probability of dengue transmission [38]. The density of the road network is also a proxy measure of the density of the human population. Cases have been found to concentrate within 500m of the road network and nearly all cases occur within a distance of 5000m [39]. Accordingly, maximum vulnerability for dengue was assumed to occur at a distance of up to 500m from the road network, then monotonically decrease to a maximum distance of 5000m, after which the location was deemed not to be vulnerable (Table 1).

### Distance from open water bodies/major rivers

Distribution of mosquitoes and dengue incidence varies in response to the distribution of open water bodies and rivers. Studies in the past have shown greater dengue incidence [40, 41] as well as higher prevalence of dengue seropositive residents in areas adjacent open water bodies [42]. Standing water formed as a consequence of rivers can act as breeding sites for mosquitoes and enhance the transmission of dengue [43]. As for the road network, maximum vulnerability was assumed to occur at a distance of up to 500m from open water, and then monotonically decrease to a maximum distance of 20,000m.

### Applying decision rules

We used the analytical hierarchy process (AHP) developed by Saaty to generate weights for each factor using a pairwise comparison matrix [15]. With this method, the relative importance of each factor was determined through pair-wise comparison by creating a ratio-matrix using a numerical scale. Each comparison determines which factor is more important and to what extent on a nine-point scale ranging from extremely less important through equal importance to extremely more important [15].

Pairs of factors were compared based on the number of studies that reported significant associations with dengue incidence/outbreaks. To this end, a literature search was conducted to extract relevant publications in the PubMed database. We used the following combination of search terms: ("dengue virus" OR dengue OR "severe dengue") AND (temperature or precipitation or rainfall or “land use/land cover” road or water or river or "population density") AND (Asia). To retrieve recent articles, the search was restricted for the time period between 2010 and 2020. Factors that had been reported more frequently were considered to be more important and rated higher in a pairwise comparison. A detailed description can be found in S2 Table. The principal eigen vector obtained from the pairwise matrix was then used to calculate best-fit of weight for each factor [44]. To calculate weights, a normalized comparison matrix was computed from the pairwise comparison matrix by dividing all elements of each column by their respective column sum. Then the algebraic average of the six columns of risk factors was computed to generate an approximate weight-vector. The weights were normalized so that its additive value was equal to one.

To measure the inconsistency associated with the pairwise comparison matrix, a consistency index (*CI*) was calculated as follows:

$$CI=\frac{\lambda_{max}-p}{(p-1)RI}$$

where, *λ_max_* is the biggest eigenvalue that can be obtained once we have its associated eigenvector, *p* is the number of columns of the matrix (i.e., risk factors) and *RI* is a random index (its value depend on the size *p*). If *CI* <0.10, then ratio indicates a reasonable or acceptable level of consistency, while *CI* ≥0.10 indicates inconsistent judgements [45].

The Pearson correlation coefficient (*r*) was calculated for each pair of factors. If *r*> 0.4, weights of both factors were reduced by 10%, while other weights increased proportionally to result in the total weight of one [46].

Weights assigned to the factors were considered statistically acceptable with the *CI* of 0.013, which is below the threshold level of 0.10 (Table 2). These weights were further adjusted based on the results of the correlation analysis (S3 Table).

**Table 2:** Pairwise comparison of matrix of criteria (risk factors) and weights in analytical hierarchy process with respect to dengue vulnerability.

Risk factors	Temperature	Rainfall	Land use	Population Density	Road network	Water bodies	Adjusted weights
Temperature	1						0.290
Rainfall	1/2	1					0.269
Land use	1/3	1/2	1				0.136
Population density	1/4	1/3	1/2	1			0.133
Road network	1/6	1/5	1/3	1/3	1		0.071
Water bodies	1/6	1/5	1/3	1/3	1/2	1	0.101

RI = 1.24, *λ_max_* = 6.084, *CI* = 0.013.

### Combination of criteria using weighted linear combination

Weighted linear combination (WLC) was used to aggregate all standardized raster layers. A low vulnerability score in one factor is compensated by a high vulnerability score in another factor. WLC has the following expression [47]:

$$S_{i}=\sum_{j=1}^{n}w_{j}x_{ij}$$

Where, *S_i_* represents the final score, *w_j_* represents the weight of the criterion *j*, and *x_ij_* represents the standardized score of the *j*^th^ criterion for the *i*th pixel. Standardization of risk factors and WLC were operationalized in the TerrSet Geospatial Monitoring and Modelling software (in short TerrSet) (Clark Labs, Worcester, MA).

### Sensitivity analysis

Sensitivity analysis was conducted by changing the MF (i.e., the structure or the shape) and weights of the risk factors. To determine the change in MF of the risk factors, following sensitivity analysis were conducted: a) All linear monotonic relationship were converted into sigmoidal relationship, b) Symmetric relationship of temperature was converted into a monotonic linear relationship and c) Rainfall was converted to a symmetric relationship from a monotonic relationship [32]. In order to assess the sensitivity of the weights, two new weights are applied to each factor by adding and subtracting 25% from the original weights [46]. We also used equal weights of 0.167 for all risk factors. Each of the newly constructed weights was individually incorporated into the model, while holding other factor weights constant. For each combination of weights obtained, a vulnerability map was created. Vulnerability estimates were extracted from both the original and new maps at the sub-district level using the *zonal statistics* tool in ArcGIS and the mean change in vulnerability was calculated. Average changes in vulnerability scores for each change in input values were assessed to determine the robustness of the model.

### Map validation

The predictive ability of the MCDA was quantified using a Receiver Operating Characteristic Curve (ROC) constructed in the R software. Predicted values were obtained by averaging the dengue vulnerability scores of all four seasons at the sub-district level. National surveillance data from NEWARS were used to identify sub-districts that have reported dengue over the past five years. These sub-district-level observations were then compared with predicted vulnerability derived from the MCDA. The area under the curve (AUC) of the ROC was used to validate the predictive ability of the MCDA map. Note, using this approach, estimates of both sensitivity and specificity are dependent on all vulnerable areas of the country having experienced dengue cases during the past five years and all areas reporting dengue having had local transmission. Both are associated with considerable uncertainty.

## Results

### Vulnerability maps for dengue

**Fig 4:**
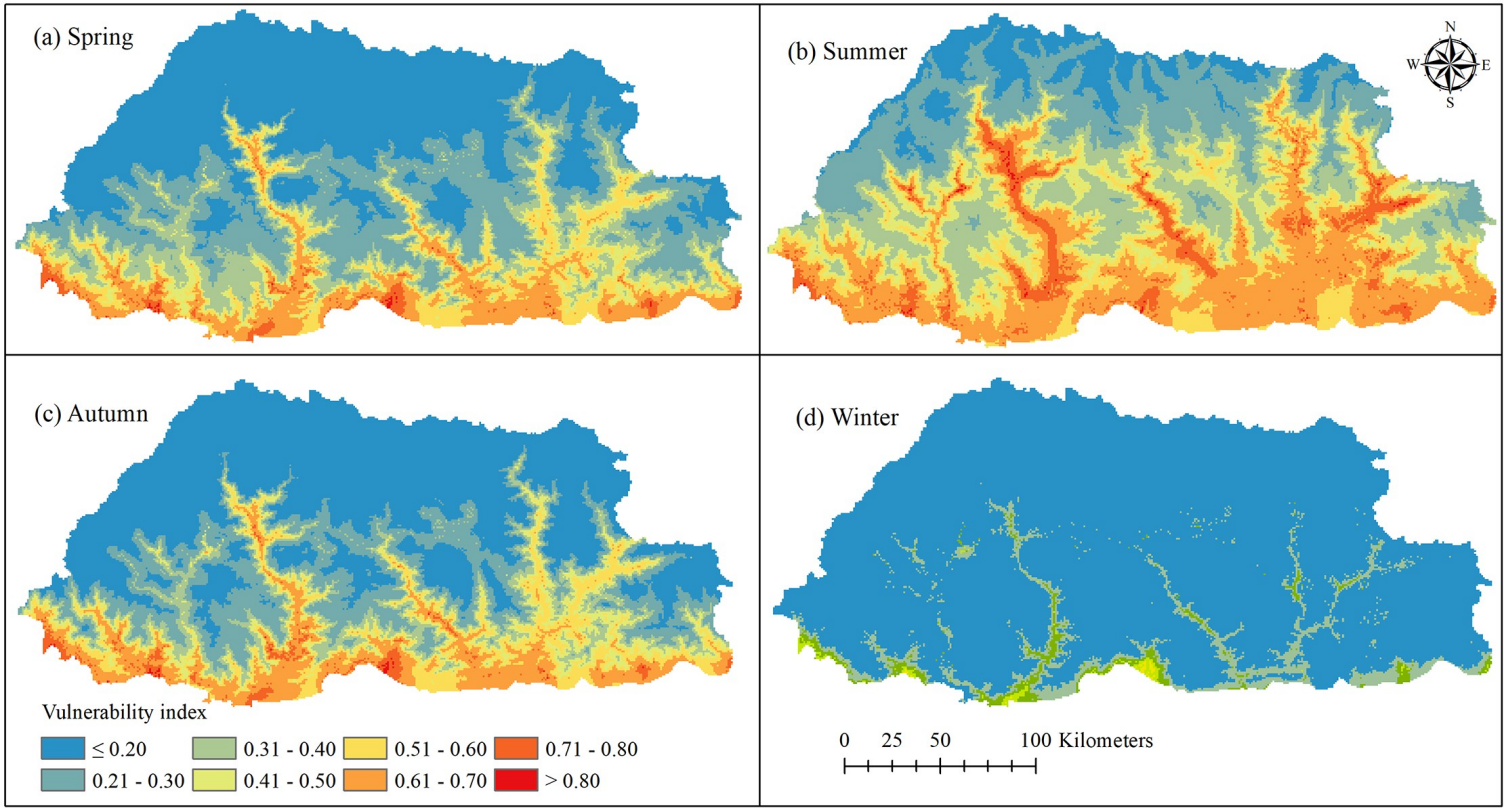
Dengue vulnerability map of Bhutan for winter, spring, summer and autumn seasons. Winter had vulnerability index upto 0.60, while all other seasons have >0.80. The geographical coverage of vulnerability index >0.80 was observed maximum in summer.

Dengue vulnerability varied by different time periods, as displayed by four different vulnerability maps covering the country of Bhutan (Fig 4). The Winter season had the lowest vulnerability for dengue up to a maximum vulnerability score of 0.6. With the onset of spring, our model projected an increase in vulnerability with extension of higher vulnerability to many geographical regions. Areas with high vulnerability (vulnerability score >0.80) were predominantly concentrated in low-lying elevations and included Chukha, Samtse, Sarpang, Dagana, Pemagatshel and Samdrup Jongkhar districts.

**Fig 5:**
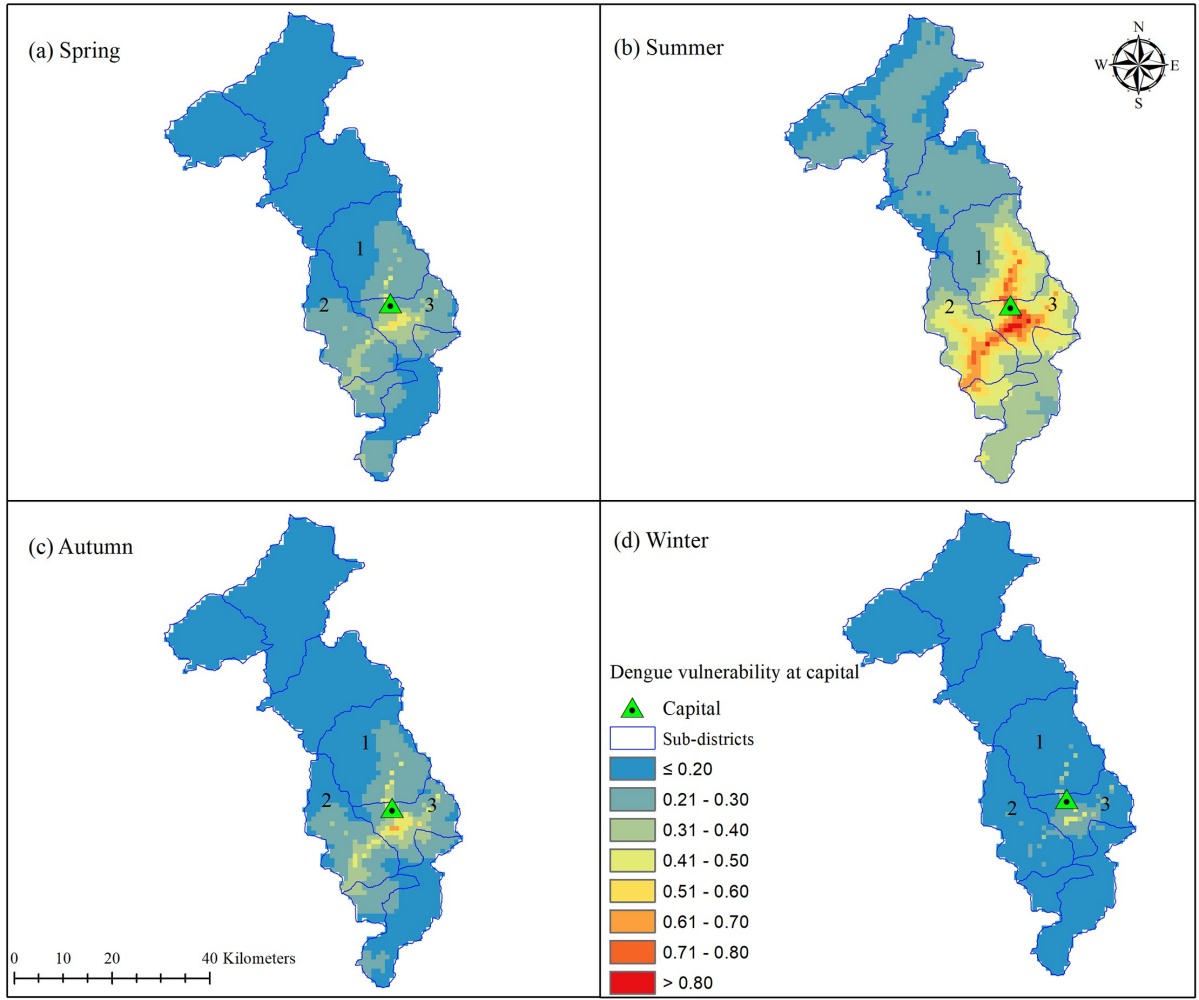
Dengue vulnerability at Thimphu sub-districts. Vulnerability index >0.80 were observed in Kawang (1), Mewang (2) and Chang (3) sub-districts during summer.

Summer had the highest vulnerability for dengue, with further expansion of vulnerable areas. Areas of vulnerability even extended to the nation’s capital, where the vulnerability score was >0.80 in three sub-districts, namely Kawang, Chang and Mewang (Fig 5). Other places with high vulnerability in summer included Wangdiphodrang, Punakha, Tsirang, Paro, Trongsa, Mongar, Trashiyangtse, Lhuentse, and Trashigang districts. In autumn, areas vulnerable for dengue started to decrease, mainly due to declining temperatures.

### Sensitivity analysis

Assuming a sigmoidal relationship between risk factors and dengue vulnerability resulted in a mean change of vulnerability estimates of 0.0067, 0.0057, 0.0018 and 0.0054 for spring, summer, autumn and winter seasons respectively. Increasing or decreasing the weights of the factors by 25% whilst holding all other weights constant resulted in a mean change in vulnerability estimates from as low as 0.0006 to 0.0335. Changing the MF of rainfall from monotonic to symmetric structure resulted in the highest overall average change in vulnerability estimates of 0.0767 (Table 3).

**Table 3:** Average change in vulnerability scores generated by subtracting scores of the original map and the newly created maps.

Weights/Structural variation	Spring		Summer	Autumn	Winter	Overall change
Temperature (variation in weight)	↑ 25%	0.0021	0.0013	0.0040	0.0048	0.0030
	↓ 25%	0.0059	0.0110	0.0045	0.0024	0.0060
Rainfall	↑ 25%	0.0098	0.0205	0.0109	0.0083	0.0124
	↓ 25%	0.0197	0.0335	0.0127	0.0065	0.0181
Land use	↑ 25%	0.0157	0.0211	0.0117	0.0051	0.0134
	↓ 25%	0.0088	0.0134	0.0125	0.0024	0.0093
Pop. Density	↑ 25%	0.0164	0.0219	0.0125	0.0058	0.0142
	↓ 25%	0.0092	0.0136	0.0129	0.0031	0.0097
Road	↑ 25%	0.0049	0.0081	0.0009	0.0019	0.0040
	↓ 25%	0.0028	0.0006	0.0010	0.0049	0.0023
Water	↑ 25%	0.0056	0.0013	0.0097	0.0144	0.0078
	↓ 25%	0.0135	0.0103	0.0098	0.0174	0.0128
Equal weights	0.0344		0.0939	0.0320	0.0671	0.0568
Linear to sigmoidal	0.0067		0.0057	0.0018	0.0054	0.0049
Rainfall symmetric	0.0109		0.2312	0.0232	0.0415	0.0767
Temperature monotonic	0.0496		0.0649	0.0529	0.0101	0.0444

* Calculated by taking arithmetic mean of all four seasons.

### Validation

The predictive performance (AUC) of the MCDA model at the sub-district level was 0.66 (95%CI: 0.57, 0.76), 0.64 (95%CI: 0.54, 0.73), 0.65 (95%CI: 0.55, 0.75), and 0.72 (95%CI: 0.64, 0.79) for spring, summer autumn and winter seasons, respectively. The overall average performance of the model was AUC = 0.66 (95% CI: 0.57, 0.75).

## Discussion

We implemented a knowledge-driven MCDA model using WLC to develop vulnerability maps for dengue, by season, in Bhutan. Areas with high vulnerability were predominantly located along the southern international boundary with India and in the river valleys projecting into the interior of the country. In agreement with the prediction map, dengue outbreaks in the past were predominantly reported from the southern border region. These areas are generally characterized by lower elevations, built-ups areas, high population densities and favourable climatic conditions for dengue transmission. People living in these areas can freely cross the international border in both directions, increasing their exposure to dengue virus infection. Other regions also reported an increasing incidence of dengue in places where there is free cross-border movement of people [48].

Vulnerability was entirely restricted to lower-elevation areas during the winter season, whereas many additional areas had enhanced risk in the other seasons, even including some higher-elevation areas. Areas found to be vulnerable during the summer season included the Thimphu (where capital city is located), Wangdiphodrang, Punakha, Tsirang, Paro, Trongsa, Mongar, Trashiyangtse, Lhuentse, and Trashigang districts. These are built-up areas, surrounded by agricultural land, with better road facilities and a high population density. High dengue incidence in built-up areas might be related to human activities such as using water tanks, plastic bottles, discarded tyres, ornamental ponds, metal drums, flower pots, buckets, trays and pots, which increases breeding habitats for immature mosquitoes [49]. Similarly, settlements with more gasoline stations and workshops, rice paddies and marshy or swampy land have been shown to have more dengue vectors [36]. As predicted by the model, a remote village in Trashiyangtse district reported its first-ever dengue outbreak in 2019. This area is located at an elevation of 915m above sea level, that few in the community expected to be vulnerable to dengue (Entomologist, Vector-borne Disease Control Programme, oral communication, 2^nd^ March 2020). The method developed here could be extended to explore the effects of future global warming given that there are no actual data currently in existence that can enable an empirical analysis.

MCDA is still growing in public health applications, although it is widely practised in other disciplines [50, 51]. Using national surveillance data, validation of our model produced an average ROC-AUCs of 0.67. This shows that our model for generating vulnerability maps is reasonably reliable without the need for additional data to refine the parameters. Sensitivity analysis revealed higher mean differences in vulnerability estimates in all the vulnerability maps when varying the shape of the MF as compared to varying the weights. This indicates the importance of defining the shape of the relationship between the risk factors and the vulnerability outcome [46]. Given that greater certainty exists around the shape of relationships than the values of the specific weightings, this increases our confidence in the model.

This study is subjected to several limitations. Factors such as literacy rate, poverty index, sanitation and hygiene could not be mapped as they were only available at coarse resolution (i.e. the district level) [52]. Up-to-date meteorological data were not available at higher resolution, and the classification of the road network into different types such as national highway or secondary road was not available. Dengue cases from the NEWARS were used to validate the model. However, those not captured by the surveillance systems who were asymptomatic or mild cases and did not seek care in public health facilities were not accounted for. Additionally, validating the vulnerability maps against data on locations where dengue had occurred doesn’t account for areas that are vulnerable for dengue but where the disease has not yet reached, or for areas where dengue was reported but no local transmission had actually occurred (i.e., all cases were imported).

Overall, our study revealed peak season and areas of vulnerability to dengue at specific locations for prioritizing intervention strategies. The findings have implications for the development of surveillance and early warning systems for dengue, allowing identification of high-risk areas to mitigate public health effects of dengue in the country.

## Supporting information

S1 Table Seasonal dengue incidence per 10,000 inhabitants between 2016 and 2019, Bhutan (Data source: NEWARS). (DOCX)

S2 Table Total number of publications reporting association between dengue incidence and selected risk factors in the Asian countries. (DOCX)

S3 Table Pearson Correlation Coefficient (r) for pair of risk factors associated with occurrence of dengue transmission. (DOCX)

S1 Fig Average annual dengue incidence per 10,000 inhabitants between in 2016–2019, Bhutan. We calculated the incidence by dividing the average number of dengue cases reported to dengue surveillance (NEWARS) between 2016 and 2019 by the average district population. (Data source: NEWARS).
(TIFF)
