# Supplementary material for: Dengue risk assessment using multicriteria decision analysis: A case study of Bhutan
Source: PLoS Negl Trop Dis. 2021 Feb 10;15(2):e0009021. doi: 10.1371/journal.pntd.0009021 (PMC7875403; doi:10.1371/journal.pntd.0009021)
Supplement: S1 Table — (DOCX) [file pntd.0009021.s001.docx]

| Season | 2016 | 2017 | 2018 | 2019 |
| --- | --- | --- | --- | --- |
| Winter (Dec - Feb) | 0.50 | 0.12 | 0.00 | 0.28 |
| Spring (Mar - May) | 0.01 | 0.18 | 0.35 | 0.65 |
| Summer (Jun - Aug) | 0.63 | 4.13 | 0.20 | 52.99 |
| Autumn (Sep - Nov) | 2.33 | 1.09 | 0.08 | 17.80 |
