# Supplementary material for: Dengue risk assessment using multicriteria decision analysis: A case study of Bhutan
Source: PLoS Negl Trop Dis. 2021 Feb 10;15(2):e0009021. doi: 10.1371/journal.pntd.0009021 (PMC7875403; doi:10.1371/journal.pntd.0009021)
Supplement: S2 Table — (DOCX) [file pntd.0009021.s002.docx]

Studies in the past have reported a statistical association between dengue incidence with temperature, rainfall, elevation, population density, land use, road network and water bodies. We used combination of search terms as described below to retrieve articles that showed association between dengue and its independent variables to calculate weights in AHP. Due to large volumes of literature available online in peer-reviewed journals, the search was confined only to Asian countries which is logically chosen to represent Bhutan. We used following combination of search terms: ((("dengue virus" OR dengue OR "severe dengue"Title OR "dengue fever" OR "dengue hemorrhagic fever"[MeSH Terms]) AND (temperature or precipitation or rainfall or “land use/land cover” road or water or river or "population density")) AND (("2010"[Date - Create]: "2020"[Date - Create]))) AND (asia[MeSH Terms]).

| Variables | Country | Total publications | Reference |
| --- | --- | --- | --- |
| Temperature | Cambodia | 1 | [1] |
|  | China | 12 | [2-13] |
|  | India | 1 | [14] |
|  | Malaysia | 1 | [15] |
|  | Pakistan | 2 | [16,17] |
|  | Philippines | 1 | [18] |
|  | Saudi Arabia | 1 | [19] |
|  | Taiwan | 4 | [20-23] |
|  | Thailand | 1 | [24] |
|  | Vietnam | 3 | [25-27] |
|  | Indonesia | 1 | [28] |
|  | Timor-Leste | 1 | [29] |
|  |  |  |  |
| Rainfall | Cambodia | 1 | [1] |
|  | China | 4 | [6,7,9,10] |
|  | Pakistan | 2 | [16,17] |
|  | Philippines | 2 | [18,30] |
|  | Taiwan | 3 | [20,21,23] |
|  | Vietnam | 2 | [25,26] |
|  | Timor-Leste | 1 | [29] |
|  |  |  |  |
| Land use | China | 5 | [2,3,9,13,31] |
|  | Pakistan | 1 | [16] |
|  | India | 1 | [14] |
|  | Taiwan | 1 | [32] |
|  | Thailand | 1 | [33] |
|  | Vietnam | 1 | [25] |
|  |  |  |  |
| Population density | China | 4 | [2,3,9,13] |
|  | Pakistan | 2 | [16,34] |
|  |  |  |  |
| Road network | China | 4 | [3,31,35,36] |
|  |  |  |  |
| Water bodies | Bangladesh | 1 | [37] |
|  | Taiwan | 1 | [38] |

**References**

1. Choi Y, Tang CS, McIver L, Hashizume M, Chan V, Abeyasinghe RR, et al. Effects of weather factors on dengue fever incidence and implications for interventions in Cambodia. BMC Public Health. 2016;16:241.

2. Zheng L, Ren HY, Shi RH, Lu L. Spatiotemporal characteristics and primary influencing factors of typical dengue fever epidemics in China. Infect Dis Poverty. 2019;8(1):24.

3. Chen Y, Zhao Z, Li Z, Li W, Li Z, Guo R, et al. Spatiotemporal Transmission Patterns and Determinants of Dengue Fever: A Case Study of Guangzhou, China. Int J Environ Res Public Health. 2019;16(14).

4. Wu X, Lang L, Ma W, Song T, Kang M, He J, et al. Non-linear effects of mean temperature and relative humidity on dengue incidence in Guangzhou, China. Sci Total Environ. 2018;628-629:766-71.

5. Jing QL, Cheng Q, Marshall JM, Hu WB, Yang ZC, Lu JH. Imported cases and minimum temperature drive dengue transmission in Guangzhou, China: evidence from ARIMAX model. Epidemiol Infect. 2018;146(10):1226-35.

6. Xiang J, Hansen A, Liu Q, Liu X, Tong MX, Sun Y, et al. Association between dengue fever incidence and meteorological factors in Guangzhou, China, 2005-2014. Environ Res. 2017;153:17-26.

7. Li C, Wang X, Wu X, Liu J, Ji D, Du J. Modeling and projection of dengue fever cases in Guangzhou based on variation of weather factors. Sci Total Environ. 2017;605-606:867-73.

8. Shen JC, Luo L, Li L, Jing QL, Ou CQ, Yang ZC, et al. The Impacts of Mosquito Density and Meteorological Factors on Dengue Fever Epidemics in Guangzhou, China, 2006-2014: a Time-series Analysis. Biomed Environ Sci. 2015;28(5):321-9.

9. Cao Z, Liu T, Li X, Wang J, Lin H, Chen L, et al. Individual and Interactive Effects of Socio-Ecological Factors on Dengue Fever at Fine Spatial Scale: A Geographical Detector-Based Analysis. Int J Environ Res Public Health. 2017;14(7).

10. Liu D, Guo S, Zou M, Chen C, Deng F, Xie Z, et al. A dengue fever predicting model based on Baidu search index data and climate data in South China. PLoS One. 2019;14(12):e0226841.

11. Zhu B, Wang L, Wang H, Cao Z, Zha L, Li Z, et al. Prediction model for dengue fever based on interactive effects between multiple meteorological factors in Guangdong, China (2008-2016). PLoS One. 2019;14(12):e0225811.

12. Gu H, Leung RK, Jing Q, Zhang W, Yang Z, Lu J, et al. Meteorological Factors for Dengue Fever Control and Prevention in South China. Int J Environ Res Public Health. 2016;13(9).

13. Yue Y, Sun J, Liu X, Ren D, Liu Q, Xiao X, et al. Spatial analysis of dengue fever and exploration of its environmental and socio-economic risk factors using ordinary least squares: A case study in five districts of Guangzhou City, China, 2014. Int J Infect Dis. 2018;75:39-48.

14. Mala S, Jat MK. Implications of meteorological and physiographical parameters on dengue fever occurrences in Delhi. Sci Total Environ. 2019;650(Pt 2):2267-83.

15. Jayaraj VJ, Avoi R, Gopalakrishnan N, Raja DB, Umasa Y. Developing a dengue prediction model based on climate in Tawau, Malaysia. Acta Trop. 2019;197:105055.

16. Mahmood S, Irshad A, Nasir JM, Sharif F, Farooqi SH. Spatiotemporal analysis of dengue outbreaks in Samanabad town, Lahore metropolitan area, using geospatial techniques. Environ Monit Assess. 2019;191(2):55.

17. Malik A, Yasar A, Tabinda AB, Zaheer IE, Malik K, Batool A, et al. Assessing spatio-temporal trend of vector breeding and dengue fever incidence in association with meteorological conditions. Environ Monit Assess. 2017;189(4):189.

18. Sumi A, Telan EF, Chagan-Yasutan H, Piolo MB, Hattori T, Kobayashi N. Effect of temperature, relative humidity and rainfall on dengue fever and leptospirosis infections in Manila, the Philippines. Epidemiol Infect. 2017;145(1):78-86.

19. Alkhaldy I. Modelling the association of dengue fever cases with temperature and relative humidity in Jeddah, Saudi Arabia-A generalised linear model with break-point analysis. Acta Trop. 2017;168:9-15.

20. Lai YH. The climatic factors affecting dengue fever outbreaks in southern Taiwan: an application of symbolic data analysis. Biomed Eng Online. 2018;17(Suppl 2):148.

21. Chuang TW, Chaves LF, Chen PJ. Effects of local and regional climatic fluctuations on dengue outbreaks in southern Taiwan. PLoS One. 2017;12(6):e0178698.

22. Yuan HY, Wen TH, Kung YH, Tsou HH, Chen CH, Chen LW, et al. Prediction of annual dengue incidence by hydro-climatic extremes for southern Taiwan. Int J Biometeorol. 2019;63(2):259-68.

23. Chang C-J, Chen CS, Tien C-J, Lu M-R. Epidemiological, clinical and climatic characteristics of dengue fever in Kaohsiung City, Taiwan with implication for prevention and control. PLoS One. 2018;13(1):e0190637.

24. Xu Z, Bambrick H, Yakob L, Devine G, Lu J, Frentiu FD, et al. Spatiotemporal patterns and climatic drivers of severe dengue in Thailand. Sci Total Environ. 2019;656:889-901.

25. Bett B, Grace D, Lee HS, Lindahl J, Nguyen-Viet H, Phuc PD, et al. Spatiotemporal analysis of historical records (2001-2012) on dengue fever in Vietnam and development of a statistical model for forecasting risk. PLoS One. 2019;14(11):e0224353.

26. Lee HS, Nguyen-Viet H, Nam VS, Lee M, Won S, Duc PP, et al. Seasonal patterns of dengue fever and associated climate factors in 4 provinces in Vietnam from 1994 to 2013. BMC Infect Dis. 2017;17(1):218.

27. Phung D, Huang C, Rutherford S, Chu C, Wang X, Nguyen M, et al. Identification of the prediction model for dengue incidence in Can Tho city, a Mekong Delta area in Vietnam. Acta Trop. 2015;141(Pt A):88-96.

28. Husnina Z, Clements ACA, Wangdi K. Forest cover and climate as potential drivers for dengue fever in Sumatra and Kalimantan 2006–2016: a spatiotemporal analysis. Trop Med Int Health. 2019;24(7):888-98.

29. Wangdi K, Clements ACA, Du T, Nery SV. Spatial and temporal patterns of dengue infections in Timor-Leste, 2005-2013. Parasit Vectors. 2018;11(1):9.

30. Iguchi JA, Seposo XT, Honda Y. Meteorological factors affecting dengue incidence in Davao, Philippines. BMC Public Health. 2018;18(1):629.

31. Qi X, Wang Y, Li Y, Meng Y, Chen Q, Ma J, et al. The Effects of Socioeconomic and Environmental Factors on the Incidence of Dengue Fever in the Pearl River Delta, China, 2013. PLoS Negl Trop Dis. 2015;9(10):e0004159.

32. Huang C-C, Tam TYT, Chern Y-R, Lung S-CC, Chen N-T, Wu C-D. Spatial Clustering of Dengue Fever Incidence and Its Association with Surrounding Greenness. Int J Environ Res Public Health. 2018;15(9):1869.

33. Sarfraz MS, Tripathi NK, Kitamoto A. Near real-time characterisation of urban environments: a holistic approach for monitoring dengue fever risk areas. International Journal of Digital Earth. 2014;7(11):916-34.

34. Atique S, Chan TC, Chen CC, Hsu CY, Iqtidar S, Louis VR, et al. Investigating spatio-temporal distribution and diffusion patterns of the dengue outbreak in Swat, Pakistan. J Infect Public Health. 2018;11(4):550-7.

35. Li Q, Ren H, Zheng L, Cao W, Zhang A, Zhuang D, et al. Ecological Niche Modeling Identifies Fine-Scale Areas at High Risk of Dengue Fever in the Pearl River Delta, China. Int J Environ Res Public Health. 2017;14(6).

36. Li Q, Cao W, Ren H, Ji Z, Jiang H. Spatiotemporal responses of dengue fever transmission to the road network in an urban area. Acta Trop. 2018;183:8-13.

37. Hashizume M, Dewan AM, Sunahara T, Rahman MZ, Yamamoto T. Hydroclimatological variability and dengue transmission in Dhaka, Bangladesh: a time-series study. BMC Infect Dis. 2012;12:98.

38. Hsueh Y-H, Lee J, Beltz L. Spatio-temporal patterns of dengue fever cases in Kaoshiung City, Taiwan, 2003–2008. Appl Geogr. 2012;34:587-94.
