# Supplementary material for: Dengue risk assessment using multicriteria decision analysis: A case study of Bhutan
Source: PLoS Negl Trop Dis. 2021 Feb 10;15(2):e0009021. doi: 10.1371/journal.pntd.0009021 (PMC7875403; doi:10.1371/journal.pntd.0009021)
Supplement: S3 Table — (DOCX) [file pntd.0009021.s003.docx]

| Risk factors | Temp | Rain | Land use | Pop. density | Road network | Water bodies |
| --- | --- | --- | --- | --- | --- | --- |
| Temp | 1 |  |  |  |  |  |
| Rain | **0.751** | 1.000 |  |  |  |  |
| Land use | **0.427** | 0.336 | 1.000 |  |  |  |
| Pop. density | 0.268 | 0.189 | **0.631** | 1.000 |  |  |
| Road network | **-0.496** | -0.205 | **-0.441** | **-0.429** | 1.000 |  |
| Water bodies | 0.018 | 0.063 | -0.133 | -0.121 | 0.039 | 1.000 |

Bolded numbers relate to multicollinearity issues with *r*>0.4000)
