# Supplementary figures and images for: Dengue risk assessment using multicriteria decision analysis: A case study of Bhutan
Source: PLoS Negl Trop Dis. 2021 Feb 10;15(2):e0009021. doi: 10.1371/journal.pntd.0009021 (PMC7875403; doi:10.1371/journal.pntd.0009021)

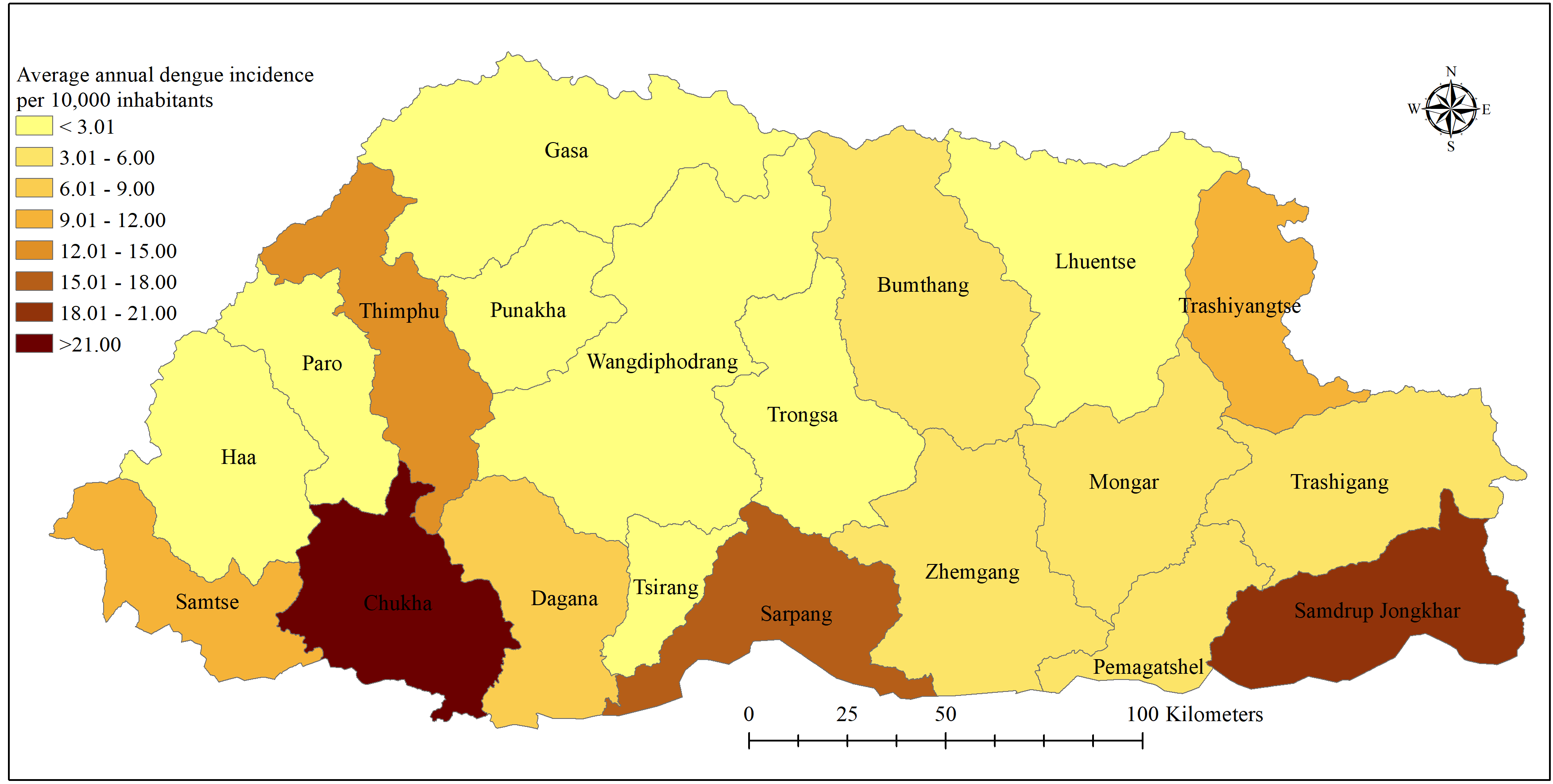

Supplement: S1 Fig — We calculated the incidence by dividing the average number of dengue cases reported to dengue surveillance (NEWARS) between 2016 and 2019 by the average district population. (Data source: NEWARS). (TIFF) [file pntd.0009021.s004.tiff]
